# 

**Published:** 1951-10

**Authors:** 


CONTENTS

| | Page |
|---|---|
| **Animal Disease in Relation to Man.** By A. M. G. CAMPBELL, D.M., F.R.C.P. | 105 |
| **The Journal: A Swan-Song.** By E. WATSON-WILLIAMS, *M.C.* | 116 |
| **The Diagnosis and Treatment of Congenital Heart Disease.** I.—DIAGNOSIS AND MEDICAL TREATMENT: By JOHN APLEY M.D., M.R.C.P. II.—SURGICAL TREATMENT: By R. MILNES WALKER, M.S., F.R.C.S. | 124 |
| **Editorial** | 139 |
| **Meetings of Societies** | 139 |
| **Local Medical Notes** | 144 |

